# Chitosan induces differential transcript usage of chitosanase 3 encoding gene (*csn3*) in the biocontrol fungus *Pochonia chlamydosporia* 123

**DOI:** 10.1186/s12864-021-08232-7

**Published:** 2022-02-05

**Authors:** Christine Sambles, Marta Suarez-Fernandez, Federico Lopez-Moya, Luis Vicente Lopez-Llorca, David J. Studholme

**Affiliations:** 1 Biosciences, University of Exeter, Exeter, UK; 2 Department of Marine Sciences and Applied Biology, Laboratory of Plant Pathology, University of Alicante, 03690 Alicante, Spain; 3 Laboratory of Plant Pathology, Multidisciplinary Institute for Environmental Studies (MIES) Ramon Margalef, University of Alicante, 03690 Alicante, Spain; 4 Centre for Plant Biotechnology and Genomics (CBGP UPM-INIA), Campus de Montegancedo, 28223 Pozuelo de Alarcon, Madrid, Spain

## Abstract

**Background:**

*Pochonia chlamydosporia* is an endophytic fungus used for nematode biocontrol that employs its cellular and molecular machinery to degrade the nematode egg-shell. Chitosanases, among other enzymes, are involved in this process. In this study, we improve the genome sequence assembly of *P. chlamydosporia* 123, by utilizing long Pacific Biosciences (PacBio) sequence reads. Combining this improved genome assembly with previous RNA-seq data revealed alternative isoforms of a chitosanase in the presence of chitosan. This study could open new insights into understanding fungal resistance to chitosan and root-knot nematode (RKN) egg infection processes.

**Results:**

The *P. chlamydosporia* 123 genome sequence assembly has been updated using long-read PacBio sequencing and now includes 12,810 predicted protein-coding genes. Compared with the previous assembly based on short reads, there are 701 newly annotated genes, and 69 previous genes are now split. Eight of the new genes were differentially expressed in fungus interactions with *Meloidogyne javanica* eggs or chitosan.

A survey of the RNA-seq data revealed alternative splicing in the *csn3* gene that encodes a chitosanase, with four putative splicing variants: *csn3_v1*, *csn3_v2*, *csn3_v3* and *csn3_v4*. When *P. chlamydosporia* is treated with 0.1 mg·mL^− 1^ chitosan for 4 days, *csn3* is expressed 10-fold compared with untreated controls. Furthermore, the relative abundances of each of the four transcripts are different in chitosan treatment compared with controls. In controls, the abundances of each transcript are nil, 32, 55, and 12% for isoforms *csn3_v1*, *csn3_v2*, *csn3_v3* and *csn3_v4* respectively. Conversely, in chitosan-treated *P. chlamydosporia*, the abundances are respectively 80, 15%, 2—3%, 2—3%. Since isoform *csn3_v1* is expressed with chitosan only, the putatively encoded enzyme is probably induced and likely important for chitosan degradation.

**Conclusions:**

Alternative splicing events have been discovered and described in the chitosanase 3 encoding gene from *P. chlamydosporia* 123. Gene *csn3* takes part in RKN parasitism process and chitosan enhances its expression. The isoform *csn3_v1* would be related to the degradation of this polymer in bulk form, while other isoforms may be related to the degradation of chitosan in the nematode egg-shell.

**Supplementary Information:**

The online version contains supplementary material available at 10.1186/s12864-021-08232-7.

## Introduction

*Pochonia chlamydosporia* (Goddard) Zare and Gams (Pc) is an endophytic fungus used for biocontrol of nematode eggs and females from plant parasitic nematodes, including root-knot nematodes (RKN) as *Meloidogyne javanica* [23] and *M. incognita* [36] and cyst nematodes such as *Globodera* spp. [31], among others. When infecting *M. javanica* eggs, Pc generates chitosan from chitin using chitin deacetylases [4]. Chitosan functional importance is reflected in the observation that Pc has more genes encoding enzymes related to chitin and chitosan degradation than any other sequenced fungus [4, 17, 18]. Chitosanases (EC 3.2.1.132, glycoside hydrolase 75, www.cazy.org) hydrolyze chitosan to oligosaccharides [30]. They have been detected in plants [13], where they have been considered as defense enzymes against pathogens [8]. Chitosanolytic enzymes are also present in soil microorganisms [1, 11, 19, 34]; most of them are chitosan-resistant, including Pc [24]. Chitosan enhances appressorium differentiation and RKN egg parasitism by Pc [6]. Genes encoding chitosanases are expressed by Pc on its own, with RKN eggs or with chitosan, but mainly with the fungus, RKN eggs and chitosan together [4, 29].

Higher eukaryotic organisms have evolved mechanisms to increase the variability of proteins that are synthesized by a cell. Alternative splicing consists of the generation of different transcripts from the same single DNA strand, resulting in proteins of different conformation and length, which usually have different activities [5]. Alternative splicing patterns can sometimes be detected in transcriptomic data, such as RNA-seq [25]. In a system with multiple experimental conditions, a distinction can be made between differential transcript expression (DTE) and differential transcript usage (DTU [27];). In DTE, it is possible to observe expression changes for at least one transcript between conditions. This implies gene overexpression or repression. In DTU, relative expression level of each isoform varies with experimental conditions. DTU implies DTE, but not vice versa [27]. DTU analyses can reveal genes that express different isoforms under given conditions. This could be related to environmental adaptation. Although alternative splicing has been found in fungi [9], it has not been widely studied. In this work we report the alternative splicing and gene expression patterns of a chitosanase in Pc isolate 123 growing with chitosan.

## Materials and methods

### Biological material

*Pochonia chlamydosporia* var. *chlamydosporia* (=*Metacordyceps chlamydosporia* var. *chlamydosporium*) isolate 123 (Pc123) (ATCC No. MYA-4875; CECT No. 20929) was selected for genome resequencing and alternative splicing analysis. Pc123 was isolated from *Heterodera avenae* infected eggs [23] in south-west Spain.

### DNA isolation and Pacific biosciences sequencing

Pc123 conidia (final concentration 10^6^ conidia·mL^− 1^) were inoculated into 250 mL flasks each containing 50 mL Potato Dextrose Broth medium (24 g·L^− 1^). Flasks were incubated at 25 °C with shaking at 120 rpm. After 5 days, mycelia were recovered by filtration through Miracloth (Calbiochem) and washed twice with sterile distilled water (SDW). DNA from Pc123 resulting fresh mycelia (ca. 0.5 g) was extracted using DNeasy Plant Mini Kit (Qiagen) following manufacturer’s instructions.

Pc123 DNA was sent to Macrogen Inc. to perform Pacific Biosciences (PacBio) sequencing. PacBio Sequel SMRT (20 Kb insert size) was used as library, with PacBio Sequel SMRT 1 cell Run as sequencing platform. Throughput was around 6–7 Gb/spl.

### Genome sequence assembly and gene-calling

We used an assembly strategy that combined de novo assembly of the new, long reads with the previously assembled and annotated genome sequence for Pc 123 (GenBank: GCA_000411695.2). First, we refined the original Pc123 annotation using RNA-seq data from Suarez-Fernandez et al. [29] with Program to Assemble Spliced Alignments (PASA; Haas et al. [10]). PacBio data were then assembled de novo with the long-read sequence assembler Canu [16]. Both assemblies were combined using RagTag, a tool for reference-guided genome assembly improvement that allows current annotation features to be preserved and updated for the new reference [3]. The full details of command lines, with parameter values and options, can be found in the Supplementary Information. BLASTx (NCBI) with standard genetic code and non-redundant protein sequences as database was used to identify homologies of selected novel genes of interest with other organisms.

### Genome annotation and alternative splicing analysis

PASA was then re-run on the new assembly to create a new annotation with updated gene models, whilst retaining all previous annotation information. Format conversion, data tidying and script preparation were done for alternative transcript usage testing.

Predicted polypeptide and transcript sequences were analysed using Pfam to detect conserved domains [22], SignalP 5.0 to detect secretion signals [2], CPC2 to assess coding potential [14] and NetSurfP-2 to detect intrinsically disordered regions (IDR) [15]. The results of these searches were combined to analyse alternative transcript usage using IsoformSwitchAnalyzeR [32, 33], which enables identification and analysis of alternative splicing and isoform switches from RNA-seq data. Full details can be found in the Supplementary Information. PacBio and RNA-seq data have been submitted to NCBI Sequence Read Archive (BioProjects PRJNA68669 and PRJNA741387, respectively).

### Identification of differentially spliced genes

After genome resequencing, RNA-seq data from Suarez-Fernandez et al. [29] were mapped against the updated genome using Salmon with a wrapper script (align_and_estimate_abundance.pl) from the Trinity software package [7]. Full details are provided in the Supplementary Information.

Suarez-Fernandez et al. [29] determined the transcriptomic effect of chitosan on Pc123 root-knot nematode parasitism. We selected Pc (control, Pc growing in minimal medium for 4 days) and PcQ (Pc growing in minimal medium amended with 0,1 mg·mL^− 1^ chitosan for 4 days) treatments from that experiment.

## Results

### PacBio sequencing reduces number of Pc123 scaffolds and predicts additional putative genes

PacBio reads (average length: 15.6 kb) were used to improve the previous assembly of the *Pochonia chlamydosporia* 123 (Pc123) genome [17] (GenBank: GCA_000411695.2). The new version of the assembly is deposited as GenBank accession GCA_000411695.4.

We transferred annotation, facilitating comparisons to previous experiments. Gene models were updated using transcriptomic data [29]. These include 20 original gene models that were merged to create 10 new genes and 69 genes that were split. Furthermore, 701 putative genes that were not previously detected have also been identified, 499 of them non-overlapping with current models. After PacBio sequencing, it was possible to reduce the number of scaffolds from 956 to 121 and that of contigs from 9087 to 8409. Finally, after this new sequencing, 12,721 genes are now predicted, which is 770 more than in the previous Pc123 genome prediction (AOSW02000000). Pc123 resequencing and genome improvement is summarized in Table 1. PacBio data have been submitted to NCBI Sequence Read Archive database with accession number SRR14907880 (BioProject PRJNA68669).

**Table 1:** Pc123 genome improvement after PacBio resequencing

	Previous assembly	Updated assembly
**Total sequence length**	42,456,589	42,540,189
**Scaffold N**_**50**_ **(bp)**	225,275	5,730,077
**Number of scaffolds**	956	121
**Number of contigs**	9087	8409
**Number of predicted genes**	**11,951 genes**	**12,721 genes** (11,951 original genes + 701 novel genes + 69 split genes)

Transcriptomic data (PRJNA741387) identifies that eight of the newly identified novel genes (*novel_gene_495_5ed78ef1*, *novel_gene_946_5ed78ef1*, *novel_gene_431_5ed78ef1*, *novel_gene_491_5ed78ef1*, *novel_gene_506_5ed78ef1*, *novel_gene_82_5ed78ef1*, *novel_gene_117_5ed78ef1*, and *novel_gene_303_5ed78ef1*) are significantly differentially expressed in at least one RNA-seq treatment; three of these are non-overlapping with current models. All of them are homologous to previously known hypothetical proteins from *P. chlamydosporia*, *Metarhizium anisopliae* or *Ustilaginoidea virens* except *novel_gene_491_5ed78ef1*, for which no significant sequence similarities can be found (Table 2). None of the split or the merged genes are significantly differentially expressed in any comparison.

**Table 2:** Eight novel genes were significantly different in at least one treatment in the RNA-seq analysis published by Suarez-Fernandez et al. [29] consisting of *Pochonia chlamydosporia* 123, root-knot nematode eggs and chitosan. Homologies for these new 8 genes were searched in the NCBI database using BLASTx

Gene name	Homologous gene accession	Organism	Protein ID (NCBI)	Query cover	Identity
*novel_gene_495_5ed78ef1*	I1G_00009526	*P. chlamydosporia 123*	RZR64940.1	27%	99.06%
*novel_gene_946_5ed78ef1*	MANI_006770	*M. anisopliae*	KFG78038.1	58%	60.81%
*novel_gene_431_5ed78ef1*	VFPPC_17841	*P. chlamydosporia 170*	XP_022285428.1	39%	98.96%
*novel_gene_491_5ed78ef1*	No significant similarity was found				
*novel_gene_506_5ed78ef1*	I1G_00009556	*P. chlamydosporia 123*	RZR63873.1	51%	57.69%
*novel_gene_82_5ed78ef1*	VFPCC_12483	*P. chlamydosporia 170*	XP_018135817.1	64%	96.30%
*novel_gene_117_5ed78ef1*	UVI_02037890	*Ustilaginoidea virens*	GAO14123.1	88%	76.54%
*novel_gene_303_5ed78ef1*	I1G_00010980	*P. chlamydosporia 123*	RZR69334.1	42%	73.04%

### Chitosan stimulates the expression of an isoform of Pc123 *cns3* gene

**Fig. 1:**
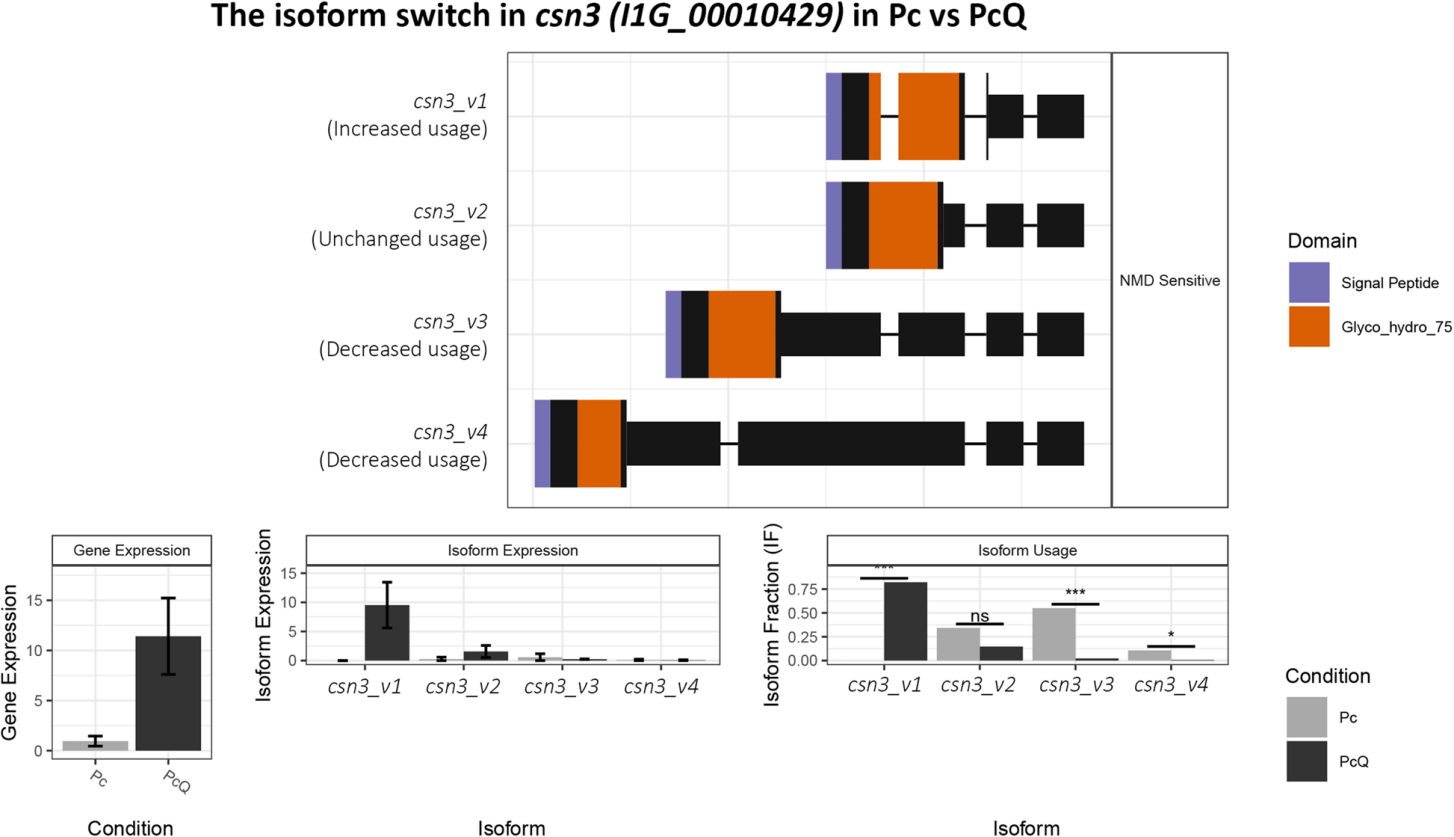
Pc123 *I1G_00010429* (RZR62940.1, *csn3*) isoforms expression with chitosan. **A**, *csn3* isoforms structure. **B**, *csn3* total gene expression with chitosan. **C**, absolute expression values of the four *csn3* isoforms. **D**, relative values of the alternative transcript usage of *csn3* with and without chitosan. Abbreviations: Pc (control without chitosan), PcQ (*P. chlamydosporia* 123 treated with 0.1 mg·mL^−1^ chitosan for 4 days [29];)

Chitosan induces alternative transcript usage in Pc123 (Fig. 1). We have found at least 20 alternatively spliced transcripts significantly expressed in Pc123 treated with chitosan (Table 3). Pc123 locus-tag I1G_00010429 or *csn3* (GenBank: RZR62940.1), which encodes chitosanase 3 [4], shows alternative transcript usage when the fungus is treated with 0.1 mg·mL^− 1^ chitosan for 4 days. We have found four isoforms for *csn3* (Fig. 1A): rna-gnl_WGS:AOSW:I1G_00010429-RA_mrna, rna-gnl_WGS:AOSW:I1G_00010429-RA_mrna.1.5ed7a624, rna-gnl_WGS:AOSW:I1G_00010429-RA_mrna.1.5ed7a624.2.5ee8159f and rna-gnl_WGS:AOSW:I1G_00010429-RA_mrna.1.5ed7a624.3.5ee8159f. We named these isoforms as *csn3_v1*, *csn3_v2*, *csn3_v3* and *csn3_v4,* respectively.

**Table 3:** Pc123 genes which show significantly expressed alternatively spliced transcripts in the presence of chitosan

Gene_ID	Condition 1	Condition 2	Gene switch q-value	Description (NCBI)
I1G_03003291	Pc	PcQ	3.93E-20	Sel1 Repeat Protein
TRINITY_DN480_c1_g1	Pc	PcQ	5.38E-17	
I1G_03009409	Pc	PcQ	3.71E-12	Hypothetical Protein
I1G_03006949	Pc	PcQ	2.32E-09	Hypothetical Protein
I1G_03002990	Pc	PcQ	5.84E-09	Hypothetical Protein
I1G_03003252	Pc	PcQ	7.35E-09	Integral Membrane Protein
**I1G_03011610**	**Pc**	**PcQ**	**1.13E-08**	**Putative Glycoside Hydrolase Family 75 Protein**
TRINITY_DN6688_c0_g1	Pc	PcQ	4.82E-07	
I1G_03004854	Pc	PcQ	7.32E-07	L-Amino-Acid Oxidase
TRINITY_DN214_c5_g1	Pc	PcQ	2.10E-06	
TRINITY_DN862_c0_g1	Pc	PcQ	4.39E-06	
I1G_03010984	Pc	PcQ	9.90E-06	Maltose Permease
TRINITY_DN10527_c0_g1	Pc	PcQ	1.10E-05	
I1G_03002977	Pc	PcQ	2.18E-05	Hypothetical Protein
TRINITY_DN2643_c0_g2	Pc	PcQ	2.27E-05	
I1G_03001483	Pc	PcQ	2.85E-05	Vacuolar Membrane Amino Acid Uptake Transporter Fnx2
TRINITY_DN6145_c0_g1	Pc	PcQ	2.86E-05	
TRINITY_DN23708_c0_g1	Pc	PcQ	2.96E-05	
TRINITY_DN12187_c0_g1	Pc	PcQ	4.61E-05	
TRINITY_DN6613_c0_g1	Pc	PcQ	5.04E-05	

In the chitosan-treated fungus Pc123 growing with chitosan, the total expression (all isoforms) of *csn3* is 10-fold higher than the total expression in the control treatment without chitosan (Fig. 1B). Adding chitosan to the medium increases *csn3_v1* isoform expression by almost 10-fold (Fig. 1C). Relative expression levels of *csn3* isoforms (Fig. 1D) in controls are ca. 55% for *csn3_v3*, ca. 32% for *csn3_v2* and ca. 12% for *csn3_v4*, while for *csn3_v1* it is nil. In the chitosan-treated fungus, *csn3_v1* isoform represents ca. 80% of gene expression, *csn3_v2* ca. 15%, and *csn3_v3* and *csn3_v4* ca. 2–3%. This means that constitutive expression of isoform *csn3_v1* is low or nil in Pc123. However, it becomes the most expressed isoform when chitosan is present in the growth medium of Pc123, suggesting that this isoform is the most efficient at degrading chitosan to chitooligosaccharides, offering a testable hypothesis for future studies.

## Discussion

In this work, we have found that chitosan induces alternative splicing events in *csn3* from *Pochonia chlamydosporia* 123. Alternative splicing occurs naturally in fungi [9, 26]. This is an important emerging issue in the regulation of fungal gene expression. Previous work demonstrates that alternative splicing events are present during root colonization by arbuscular mycorrhizal fungi [37] and plant infection by *Sclerotinia sclerotiorum* [12]. Therefore, fungi activate alternative splicing processes under different conditions to adapt to a changing environment. This suggests alternative splicing events may be related to epigenetics [20] and it may be the environment that determines relative levels of transcript expression. It has been shown that fungi, as well as animals and plants, are highly dependent on epigenetics [21]. Therefore, studying fungal alternative splicing is a promising avenue for future studies related to the effect of environment on gene expression. Understanding the mechanism by which a fungus generates a series of transcripts from a single DNA molecule could help to unravel how it responds to a stimulus. Thus, not taking into account transcriptional variants in RNA-seq analyses may lead to loss of knowledge of alternative transcript usage in key genes. This could lead to incomplete conclusions. RNA-seq analyses should therefore consider splicing variants [28]. Previous studies of alternative splicing in chitosanases demonstrate that differential expression, as in the case of *csn3*, is a common event [35]. Based on that work, we believe that *csn3* isoforms could have different functions or even locations [35]. On the other hand, *Pochonia chlamydosporia* 123 (Pc123) is known to have high chitosanolytic activity [24] due to its high content in chitosanases [4]. Future work will extend alternative transcript usage analyses to the rest of Pc123 chitosanases encoded in its genome in order to determine whether they also undergo alternative splicing events. Aranda-Martinez et al. [4] show that *csn3* is induced six-fold during Pc123 RKN parasitism. Besides, expression value of *csn3* when Pc123 infects RKN eggs in a medium amended with chitosan almost doubles the value respect to only-chitosan treatment [29]. This suggests *csn3* is one of the key genes that take part in RKN parasitism and chitosan enhances its expression. The chitosan-promoting isoform may be related to the degradation of this polymer in bulk form, while other isoforms may be related to the degradation of chitosan in the fungal or RKN egg wall. This could open new insights into understanding fungal resistance to chitosan and RKN egg infection processes.

## Supplementary Information

**Additional file 1.**

## Data Availability

PacBio and RNA-seq data have been submitted to NCBI SRA database (BioProjects PRJNA68669 and PRJNA741387, respectively). Both can be accessed following these links: PacBio data: https://www.ncbi.nlm.nih.gov/Traces/study/?acc=PRJNA68669&o=acc_s%3Aa RNA-seq data: https://www.ncbi.nlm.nih.gov/Traces/study/?acc=PRJNA741387&o=acc_s%3Aa
