# Supplementary material for: Chitosan induces differential transcript usage of chitosanase 3 encoding gene (csn3) in the biocontrol fungus Pochonia chlamydosporia 123
Source: BMC Genomics. 2022 Feb 5;23:101. doi: 10.1186/s12864-021-08232-7 (PMC8817618; doi:10.1186/s12864-021-08232-7)
Supplement: Supplementary file 1 — Additional file 1. [file 12864_2021_8232_MOESM1_ESM.pdf]

Chitosan RNA-Seq Methods

Christine Sambles

2019-10-22

Contents

1 Introduction 1

1.1 Brief description of Project: 1

2 QC 2

2.1 Software used: 2

2.2 Workflow: 3

2.3 Summary of QC results: 3

3 Genome-guided assembly 4

3.1 Software used: 4

3.2 Workflow 1: HISAT2/StringTie: 5

3.3 Workflow 2: Genome-guided Trinity assembly 7

3.4 Summary of alignment: 10

4 *De novo* assembly 10

4.1 Software used: 10

4.2 Align reads to genome with STAR aligner: 11

4.3 *De novo* transcriptome assembly with Trinity 12

5 Transcriptome consolidation 12

5.1 Software used: 12

5.2 Build a Comprehensive Transcriptome Database Using Genome-guided and *de novo* RNA-Seq Assembly 12

6 Quantification using Salmon 13

6.1 Software used: 13

1 Introduction

1.1 Brief description of Project:

We designed an experiment in which we want to evaluate the effect of chitosan on Pc (*Pochonia chlamydosporia*) during root-knot nematode (RKN) egg infection for 4 days. We have 4 treatments with 3 replicates each.
Experimental groups:

- Pc (Pc)
- Pc+chitosan (PcQ)
- Pc+RKN eggs (PcRKN)
- Pc+RKN eggs+chitosan (PcRKNQ)

2 QC

2.1 Software used:

- FASTQC / FASTQ-screen (QC)
  - FastQC aims to provide a simple way to do some quality control checks on raw sequence data coming from high throughput sequencing pipelines.
  - FastQ-screen is is a simple application which allows you to search a large sequence dataset against a panel of different databases to build up a picture of where the sequences in your data originate.
- Trimmomatic (trim/filter)
  - Trimmomatic is a flexible read trimming tool for Illumina NGS data. Used adapters file from BBmap.
- BUSCO/DETONATE/TransRate/rnaQUAST
  - BUSCO provides quantitative measures for the assessment of genome assembly, gene set, and transcriptome completeness, based on evolutionarily-informed expectations of gene content from near-universal single-copy orthologs selected from OrthoDB v9.

2.2 Workflow:

1. Initial QC: **FASTQC**
   - Using FASTQC v. 0.11.8
   - Outputs are available in QC folder.
2. Initial QC: **FASTQ Screen**
   - FASTQ-screen with additional RKN and *Pochonia chlamydosporia* libraries.
   - Outputs are available in QC folder.

```
fastq_screen --aligner bowtie2  PcQ_1_1.fastq
```

3. **Trimming reads**

2.2.1 e.g. For PcQ1:

```
trimmomatic-0.38.jar PE -threads 8 -phred33 -summary PcQ_1_v2_statsSummary.txt \
PcQ_1_1_v2.fastq.gz PcQ_1_2_v2.fastq.gz PcQ_1_1_v2_TR.fastq PcQ_1_1_v2_TRUP.fastq \
PcQ_1_2_v2_TR.fastq PcQ_1_2_v2_TRUP.fastq ILLUMINACLIP:adapters.fa:2:30:10 \
LEADING:3 TRAILING:3 SLIDINGWINDOW:4:15 MINLEN:80
```

2.2.2 This will perform the following:

- Remove adapters (ILLUMINACLIP:adapters.fa:2:30:10)
- Remove leading low quality or N bases (below quality 3) (LEADING:3)
- Remove trailing low quality or N bases (below quality 3) (TRAILING:3)
- Scan the read with a 4-base wide sliding window, cutting when the average quality per base drops below 15 (SLIDINGWINDOW:4:15)
- Drop reads below the 80 bases long (MINLEN:80)

2.3 Summary of QC results:

Group	FASTQC	FASTQ.screen	Trimmomatic
PcQ	ok	ok	**0.9655**
PcQ	ok	ok	**0.9644**
PcQ	ok	ok	**0.9668**
PcRKNQ	ok	NA	**0.9687**
PcRKNQ	ok	ok	**0.9695**
PcRKNQ	ok	ok	**0.9713**
PcRKN	ok	ok	**0.9642**
PcRKN	ok	ok	**0.9709**
PcRKN	ok	ok	**0.9625**
Pc	ok	ok	**0.9609**
Pc	moderate quality	moderate quality	**0.9687**
Pc	ok	ok	**0.9658**

3 Genome-guided assembly

3.1 Software used:

- Trinity (Assembly)
  - Trinity assembles transcript sequences from Illumina RNA-Seq data.
- HISAT2 /STAR (splice-aware align)
  - HISAT2 is a fast and sensitive alignment program for mapping next-generation sequencing reads (both DNA and RNA) to a population of human genomes (as well as to a single reference genome).
  - STAR is an ultrafast universal RNA-seq aligner.
- StringTie (novel transcript discovery)
  - StringTie is used for transcript assembly and quantification for RNA-Seq.
- IGV
- gffread
- samtools
- gffcompare

3.2 Workflow 1: HISAT2/StringTie:

1. Alignment to genome: **HISAT2**

```
hisat2 -p 16 --min-intronlen 40 --max-intronlen 2000 --dta -x PC123_ss_exons \
-1 /gpfs/ts0/home/cms209/Research_Project-181602/trimmed/PcQ_1_1_ v2_TR.fastq \
-2 /gpfs/ts0/home/cms209/Research_Project-181602/trimmed/PcQ_1_2_v2_TR.fastq \
-U /gpfs/ts0/home/cms209/Research_Project-181602/trimmed/PcQ_1_1_v2_TRUP.fastq,
/gpfs/ts0/home/cms209/Research_Project-181602/trimmed/PcQ_1_2_v2_TRUP.fastq \
-S PcQ_1_v2_trimmed_PC123_ss_exons.sam

samtools view -Sb -@ 16 PcQ_1_v2_trimmed_PC123_ss_exons.sam | samtools sort -@ 16 \
-o PcQ_1_v2_tr_PC123_se_sorted.bam -
```

2. Novel transcript discovery: **StringTie**

```
stringtie Pc_1_tr_PC123_se_sorted.bam  \
-p 16 \
--rf \
-l Pc1_HISAT_PC123 \
-o Pc1_HISAT_PC123_stringtie.gtf \
-B -G  ../../genomes/123/GCA_000411695.2_PcB1v2_genomic.gff \
-A   Pc1_HISAT_PC123_stringtie_gene_abund.tab \
-C   Pc1_HISAT_PC123_stringtie_cov_refs.gtf

# Then run StringTie with --merge in order to generate a non-redundant set of transcripts
# observed in all the RNA-Seq samples assembled previously.
stringtie --merge \
-p 16 \
-G /gpfs/ts0/home/cms209/Research_Project-181602/genomes/123/
GCA_000411695.2_PcB1v2_genomic.gff \
-o PC123_stringtie_merged.gtf \
-l PC123m /gpfs/ts0/home/cms209/Research_Project-181602/genome_guided/stringtie_merge/
GTF_list_hisat.txt

gffread -w   PC123_stringtie_merged_transcripts_v2.fa \
-g   ~/cms209/projects/banana/endophytes/pochonia/ncbi_genome_123/
GCA_000411695.2_PcB1v2_genomic.fna PC123_stringtie_merged_v2.gtf

# e.g.
stringtie Pc_1_tr_PC123_se_sorted.bam  \
-p 16 \
--rf \
-l Pc1_HISAT_PC123_p2 \
-o Pc1_HISAT_PC123_stringtie _p2 .gtf \
-b Pc1_HISAT_PC123_p2 \
-G PC123_stringtie_merged_v2.gtf  \
-A  Pc1_HISAT_PC123_stringtie_gene_abund_p2.tab \
-C  Pc1_HISAT_PC123_stringtie_cov_refs _p2 .gtf
```

3. **Compare** GFFs from Pc123 and new HISAT2/StringTie transcriptome

```
# Compare GFFs:
gffcompare \-r  ~/cms209/projects/banana/endophytes/pochonia/ncbi_genome_123/
GCA_000411695.2_PcB1v2_genomic.gtf -o gffcompare_v2   PC123_stringtie_merged_v2.gtf

cat gffcompare_v2.stats

#= Summary for dataset: PC123_stringtie_merged_v2.gtf
#     Query mRNAs :   39008 in   20502 loci  (29820 multi-exon transcripts)
#            (8537 multi-transcript loci, ~1.9 transcripts per locus)
# Reference mRNAs :   11961 in   11961 loci  (8942 multi-exon)
# Super-loci w/ reference transcripts:    11133
#-----------------| Sensitivity | Precision  |
        Base level:   100.0     |    43.2    |
        Exon level:   100.0     |    37.5    |
      Intron level:   100.0     |    48.5    |
Intron chain level:   100.0     |    30.0    |
  Transcript level:   100.0     |    30.7    |
       Locus level:   100.0     |    54.3    |

     Matching intron chains:    8942
       Matching transcripts:   11961
              Matching loci:   11961

          Missed exons:       0/34520   (  0.0%)
           Novel exons:   36192/93326   ( 38.8%)
        Missed introns:       0/22559   (  0.0%)
         Novel introns:   19592/46538   ( 42.1%)
           Missed loci:       0/11961   (  0.0%)
            Novel loci:    9365/20502   ( 45.7%)


 Total union super-loci across all input datasets: 20502
39008 out of 39008 consensus transcripts written in gffcompare_v2.annotated.gtf
(0 discarded as redundant)
```

4. **StringTie** pass 2 and **GFF3 comparison** with Pc123

```
#e.g.
stringtie Pc_1_tr_PC123_se_sorted.bam  \
-p 16 --rf \
-l Pc1_HISAT_PC123_p2 \
-o Pc1_HISAT_PC123_p2_stringtie.gtf \
-b Pc1_HISAT_PC123_p2 \
-G  PC123_stringtie_merged_v2.gtf \
-A   Pc1_HISAT_PC123_p2_stringtie_gene_abund.tab \
-C   Pc1_HISAT_PC123_p2_stringtie_cov_refs.gtf


stringtie --merge \
-p 16 \
-G /gpfs/ts0/home/cms209/Research_Project-181602/genomes/123/
GCA_000411695.2_PcB1v2_genomic.gff \
-o PC123_stringtie_merged_v2_p2.gtf \
```

```
-l PC123mv2 stringtie_merge_list_p2.txt


gffcompare -r  ~/cms209/projects/banana/endophytes/pochonia/ncbi_genome_123/
GCA_000411695.2_PcB1v2_genomic.gtf -o gffcompare_v2_p2 PC123_stringtie_merged_v2_p2.gtf


# gffcompare v0.10.6 | Command line was:
# gffcompare -r /home/cms209/cms209/projects/banana/endophytes/pochonia/ncbi_genome_123/
# GCA_000411695.2_PcB1v2_genomic.gtf -o gffcompare_v2_p2 PC123_stringtie_merged_v2_p2.gtf
#


#= Summary for dataset: PC123_stringtie_merged_v2_p2.gtf
#     Query mRNAs :   39166 in   20274 loci  (30127 multi-exon transcripts)
#            (8443 multi-transcript loci, ~1.9 transcripts per locus)
# Reference mRNAs :   11961 in   11961 loci  (8942 multi-exon)
# Super-loci w/ reference transcripts:    11085
#-----------------| Sensitivity | Precision  |
        Base level:   100.0     |    43.1    |
        Exon level:   100.0     |    37.3    |
      Intron level:   100.0     |    47.9    |
Intron chain level:   100.0     |    29.7    |
  Transcript level:   100.0     |    30.5    |
       Locus level:   100.0     |    54.7    |

     Matching intron chains:    8942
       Matching transcripts:   11961
              Matching loci:   11961

          Missed exons:       0/34520   (  0.0%)
           Novel exons:   36277/93830   ( 38.7%)
        Missed introns:       0/22559   (  0.0%)
         Novel introns:   20172/47144   ( 42.8%)
           Missed loci:       0/11961   (  0.0%)
            Novel loci:    9185/20274   ( 45.3%)
```

3.3 Workflow 2: Genome-guided Trinity assembly

5. Merge aligned files and perform **Genome-guided assembly using Trinity**

```
# Merge all BAM files from STAR alignments (see de-novo)
samtools merge -@ 16 STARPC123_v2.bam
STARPC123_PcRKNQ_v2_1_2passAligned.sortedByCoord.out.bam
STARPC123_PcRKNQ_3_2passAligned.sortedByCoord.out.bam
STARPC123_PcRKNQ_2_2passAligned.sortedByCoord.out.bam
STARPC123_PcRKN_3_2passAligned.sortedByCoord.out.bam
STARPC123_PcRKN_2_v2_2passAligned.sortedByCoord.out.bam
STARPC123_PcRKN_1_2passAligned.sortedByCoord.out.bam
STARPC123_PcQ_3_2passAligned.sortedByCoord.out.bam
STARPC123_PcQ_2_v2_2passAligned.sortedByCoord.out.bam
STARPC123_PcQ_1_v2_2passAligned.sortedByCoord.out.bam
```

```
STARPC123_Pc_3_2passAligned.sortedByCoord.out.bam
STARPC123_Pc_2_2passAligned.sortedByCoord.out.bam
STARPC123_Pc_1_2passAligned.sortedByCoord.out.bam

# Run Genome-guided Trinity
~/bin/Trinity --monitoring --max_memory 10G \
--genome_guided_bam /gpfs/ts0/projects/Research_Project-181602/STAR_incl_unmapped/pass2v2
/bam/STARPC123_v2.bam \
--genome_guided_max_intron 3000 \
--jaccard_clip \
--SS_lib_type RF \
--CPU 16 \
--output Trinity_STAR_gg
```

Produced 69,927 transcripts.

6. **Align transcripts** from genome-guided Trinity assembly to Pc123 genome:

```
gmap -d PC123 -D ./ -f 2 /mnt/bio-tarako-home/cms209/projects/banana/endophytes/pochonia/
chitosan/assembly/genome_guided/Trinity_gg/Trinity_gg_v2/trinitygg/Trinity_STAR_gg/
Trinity-GG_v2.fasta > Trinity-GG_v2.PC123_gene.gff3
```

7. **Compare** genome-guided Trinity GFF3 with Pc123

```
gffcompare -r  ~/cms209/projects/banana/endophytes/pochonia/ncbi_genome_123/
GCA_000411695.2_PcB1v2_genomic.gtf -o gffcompare_Tringg_v2 Trinity-GG_v2.PC123_gene.gff3


# gffcompare v0.10.6 | Command line was:
#gffcompare -r /home/cms209/cms209/projects/banana/endophytes/pochonia/ncbi_genome_123/
#GCA_000411695.2_PcB1v2_genomic.gtf -o gffcompare_Tringg_v2 Trinity-GG_v2.PC123_gene.gff3
#


#= Summary for dataset: Trinity-GG_v2.PC123_gene.gff3
#     Query mRNAs :   71178 in   42614 loci  (32783 multi-exon transcripts)
#            (11195 multi-transcript loci, ~1.7 transcripts per locus)
# Reference mRNAs :   11961 in   11961 loci  (8942 multi-exon)
# Super-loci w/ reference transcripts:    10683
#-----------------| Sensitivity | Precision  |
        Base level:    94.9     |    29.3    |
        Exon level:    30.7     |     9.6    |
      Intron level:    70.4     |    39.7    |
Intron chain level:    42.2     |    11.5    |
  Transcript level:    37.7     |     6.3    |
       Locus level:    37.7     |    10.5    |

     Matching intron chains:    3777
       Matching transcripts:    4515
              Matching loci:    4515

          Missed exons:    1444/34520   (  4.2%)
```

```
          Novel exons:   67395/111766  ( 60.3%)
        Missed introns:    3763/22559   ( 16.7%)
         Novel introns:   20503/40072   ( 51.2%)
           Missed loci:     299/11961   (  2.5%)
            Novel loci:   30363/42614   ( 71.3%)


 Total union super-loci across all input datasets: 41291
71178 out of 71178 consensus transcripts written in
gffcompare_Tringg_v2.annotated.gtf (0 discarded as redundant)
```

8. **Compare** GFF3s from HISAT2/StringTie and genome-guided Trinity

```
gffcompare -r   Trinity-GG_v2.PC123_gene.gff3  -o gffcompare_TrinGGvStringTiev2p2 \
PC123_stringtie_merged_v2_p2.gtf


# gffcompare v0.10.6 | Command line was:
# gffcompare -r Trinity-GG_v2.PC123_gene.gff3 -o gffcompare_TrinGGvStringTiev2p2
# PC123_stringtie_merged_v2_p2.gtf
#


#= Summary for dataset: PC123_stringtie_merged_v2_p2.gtf
#     Query mRNAs :   39166 in   20274 loci  (30127 multi-exon transcripts)
#            (8443 multi-transcript loci, ~1.9 transcripts per locus)
# Reference mRNAs :   69044 in   42614 loci  (31373 multi-exon)
# Super-loci w/ reference transcripts:    17345
#-----------------| Sensitivity | Precision  |
        Base level:    67.1     |    93.8    |
        Exon level:    26.6     |    33.3    |
      Intron level:    70.2     |    59.7    |
Intron chain level:    31.3     |    32.6    |
  Transcript level:    18.2     |    32.1    |
       Locus level:    25.6     |    53.6    |

     Matching intron chains:    9826
       Matching transcripts:   12554
              Matching loci:   10901

          Missed exons:   28717/109428  ( 26.2%)
           Novel exons:    2838/93830   (  3.0%)
        Missed introns:    8894/40072   ( 22.2%)
         Novel introns:   10300/47144   ( 21.8%)
           Missed loci:   17159/42614   ( 40.3%)
            Novel loci:     715/20274   (  3.5%)


 Total union super-loci across all input datasets: 18372
39166 out of 39166 consensus transcripts written in
gffcompare_TrinGGvStringTiev2p2.annotated.gtf (0 discarded as redundant)
```

3.4 Summary of alignment:

```
## Warning: package 'dplyr' was built under R version 3.6.1
```

```
## Warning: package 'kableExtra' was built under R version 3.6.1
```

Table 1: (#tab:Import summary table)Summary table

		against Pc123 genome				
Sample	Group	HISAT2	STAR.1.unique	STAR.1.multi	STAR.2.unique	STAR.2.multi
PcQ.1.v2	PcQ	71.8%	67.9%	0.0700%	67.9%	0.0900%
PcQ.2.v2	PcQ	72.1%	64.7%	0.0800%	64.8%	0.0800%
PcQ.3	PcQ	72.0%	69.9%	0.1000%	69.9%	0.1300%
PcRKNQ.1.v2	PcRKNQ	61.8%	54.2%	0.0600%	54.2%	0.0700%
PcRKNQ.2	PcRKNQ	75.0%	73.5%	0.1000%	73.5%	0.1200%
PcRKNQ.3	PcRKNQ	68.4%	66.7%	0.1000%	66.7%	0.1400%
PcRKN.1	PcRKN	71.3%	68.6%	0.0800%	68.6%	0.0900%
PcRKN.2.v2	PcRKN	66.4%	64.7%	0.0800%	62.5%	0.0800%
PcRKN.3	PcRKN	68.0%	65.9%	0.1000%	66.0%	0.1300%
Pc.1	Pc	68.3%	62.6%	0.0500%	62.6%	0.0600%
Pc.2	Pc	56.6%	54.3%	0.0800%	54.3%	0.0900%
Pc.3	Pc	71.9%	70.3%	0.1000%	70.3%	0.1300%

4 *De novo* assembly

4.1 Software used:

- Trinity (Assembly)
  - Trinity assembles transcript sequences from Illumina RNA-Seq data.
- Salmon (Quantification)
  - Salmon is a tool for quantifying the expression of transcripts using RNA-seq data.
- DESeq2 (DGE analysis)
  - DESeq2 is used for Differential gene expression analysis based on the negative binomial distribution.
- HISAT2 /STAR (splice-aware align)
  - HISAT2 is a fast and sensitive alignment program for mapping next-generation sequencing reads (both DNA and RNA) to a population of human genomes (as well as to a single reference genome).
  - STAR is an ultrafast universal RNA-seq aligner.
- StringTie (novel transcript discovery)
  - StringTie is used for transcript assembly and quantification for RNA-Seq.
- PASA
- Trinotate
- DETONATE evaluates de novo transcriptome assemblies from RNA-Seq data.
- Transrate is software for de-novo transcriptome assembly quality analysis.

- rnaQUAST is a tool for evaluating RNA-Seq assembly quality and benchmarking transcriptome assemblers using reference genome and gene database.
- IGV
- gffread
- SRA toolkit
- samtools
- gffcompare

4.2 Align reads to genome with STAR aligner:

```
#e.g.
# Pass 1:
mkdir ./STAR123_1

# Generate genome files for pass 1:
~/bin/STAR \
--runThreadN 16 \
--runMode genomeGenerate \
--genomeDir ./STAR123_1/ \
--genomeFastaFiles ./genomes/123/GCA_000411695.2_PcB1v2_genomic.fna  \
--sjdbGTFfile ./genomes/123/GCA_000411695.2_PcB1v2_genomic.gtf

# Run alignment pass 1:
~/bin/STAR \
--runThreadN 16 \
--genomeDir ./STAR123_1 \
--outFileNamePrefix STARPC123_PcQ_1  \
--outSAMtype BAM SortedByCoordinate \
--outSAMunmapped Within \
--outSAMstrandField intronMotif \
--readFilesIn ./trimmed/PcQ_1_1_TR.fastq ./trimmed/PcQ_1_2_TR.fastq

# Pass 2:
mkdir ./STAR123_2

# Generate genome files for pass 2:
STAR --runThreadN 16 \
--runMode genomeGenerate \
--genomeDir  ./STAR123_2 \
--genomeFastaFiles  ./genomes/123/GCA_000411695.2_PcB1v2_genomic.fna  \
--sjdbGTFfile  ./genomes/123/GCA_000411695.2_PcB1v2_genomic.gtf  \
--sjdbFileChrStartEnd STARPC123_PcQ_2SJ.out.tab STARPC123_PcRKNQ_3SJ.out.tab
STARPC123_Pc_2SJ.out.tab STARPC123_PcQ_3SJ.out.tab STARPC123_PcRKN_2SJ.out.tab
STARPC123_Pc_3SJ.out.tab STARPC123_PcRKNQ_1SJ.out.tab  STARPC123_PcRKN_3SJ.out.tab
STARPC123_PcQ_1SJ.out.tab  STARPC123_PcRKNQ_2SJ.out.tab  STARPC123_Pc_1SJ.out.tab

# Run alignment pass 2:
STAR --runThreadN 16 \
--genomeDir ./STAR123_2 \
--outFileNamePrefix STARPC123_PcQ_1_2pass  \
--outSAMtype BAM SortedByCoordinate \
--outSAMunmapped Within \
```

```
--outSAMstrandField intronMotif \
--readFilesCommand zcat \
--readFilesIn ./trimmed/PcQ_1_1_TR.fastq.gz ./trimmed/PcQ_1_2_TR.fastq.gz
```

4.3 *De novo* transcriptome assembly with Trinity

```
~/bin/Trinity --monitoring \
--no_distributed_trinity_exec \
--grid_exec "/gpfs/ts0/home/cms209/HpcGridRunner-1.0.2/hpc_cmds_GridRunner.pl --grid_conf
/gpfs/ts0/home/cms209/HpcGridRunner-1.0.2/PBS.conf -c" \
--grid_node_CPU 16 \--grid_node_max_memory 10G \
--seqType fq --SS_lib_type RF \
--max_memory 10G \
--jaccard_clip \
--CPU 16 \
--samples_file Trinity_samples.txt \
--output Trinity_dn
```

5 Transcriptome consolidation

5.1 Software used:

- PASA
  - a eukaryotic genome annotation tool
  - Allows a hybrid approach to transcript reconstruction using genome-guided and *de novo* RNA-Seq assemblies to generate a comprehensive transcript database

5.2 Build a Comprehensive Transcriptome Database Using Genome-guided and *de novo* RNA-Seq Assembly

1. Copy the config file to database directory

```
cp /home/cms209/cms209/software/PASApipeline/pasa_conf/pasa.alignAssembly.Template.txt
./alignAssembly.config
```

2. Change DATABASE to /tmp/sample__mydb__pasa

3. Concatenate the Trinity.fasta and Trinity.GG.fasta files into a single ‘transcripts.fasta’ file.

```
cat Trinity.fasta Trinity-GG.fasta Trinity_unmapped_ed.fasta  > Trinall.fasta
cat Trinity.fasta Trinity-GG.fasta Trinity_unmapped_ed.fasta spades.fasta> transcripts.fasta
```

4. Create a file containing the list of transcript accessions that correspond to the Trinity *de novo* assembly (full *de novo*, not genome-guided).

```
/home/cms209/cms209/software/PASApipeline/misc_utilities/accession_extractor.pl <Trinity.fasta>
tdn.accs
```

5. Run PASA using RNA-Seq related options as described in the section above, but include the parameter setting '–TDN tdn.accs', -C (create) and -R (run):

```
nohup /home/cms209/cms209/software/PASApipeline/Launch_PASA_pipeline.pl \
-c alignAssembly.config \
-C -R \
-g /home/cms209/cms209/projects/banana/endophytes/pochonia/ncbi_genome_123/
GCA_000411695.2_PcB1v2_genomic.fna \
--ALIGNERS blat,gmap \
-t Trinall.fasta \
--TDN tdn.accs \
--trans_gtf  PC123_stringtie_merged_v2_p2.gtf \
--transcribed_is_aligned_orient \
--stringent_alignment_overlap 30.0 \
--CPU 8 &> pasa.nohup &
```

6. After completing the PASA alignment assembly, generate the comprehensive transcriptome database via:

```
/home/cms209/cms209/software/PASApipeline/scripts/build_comprehensive_transcriptome.dbi \
-c  alignAssembly.config \
-t Trinall.fasta \
--min_per_ID 95 \
--min_per_aligned 30
```

6 Quantification using Salmon

6.1 Software used:

- Trinity (Assembly)
  - Trinity assembles transcript sequences from Illumina RNA-Seq data.
- Salmon
  - a tool for quantifying the expression of transcripts using RNA-seq data.

1. Use Trinity script to do gene-level analysis using Salmon:

```
/bioinformatics/christine/software/trinityrnaseq-Trinity-v2.8.5/util/align_and_estimate_abundance.pl
--transcripts compreh_init_build.fasta --seqType fq --samples_file Trinity_samples.txt --est_method
salmon --output_dir ./salmon --SS_lib_type RF --thread_count 16 --gene_trans_map
compreh_init_build.geneToTrans_mapping
```
